# Large lumbosacral schwannoma in a young female- a case report from Afghanistan

**DOI:** 10.1016/j.amsu.2021.102986

**Published:** 2021-10-30

**Authors:** Sayed Hanif Monawary, Shafi Ullah Zahid, Kalimullah Wardak, Kiran Shafiq Khan, Irfan Ullah, Zohaib Yousaf

**Affiliations:** a Department of Neurosurgery, Jamhuriat Hospital, Kabul, Afghanistan; b Department of Orthopedics and Traumatology, Wazir Mohammad Akbar Khan (WMAK) Hospital, Kabul, Afghanistan; c Dow Medical College, Dow University of Health Sciences, Karachi, 74200, Pakistan; d Kabir Medical College, Gandhara University, Peshawar, 25000, Pakistan; e Hamad Medical Corporation, Doha, Qatar

**Keywords:** Schwannomas, Benign tumor, Lumbar spine, Nerve sheath tumor, Laminectomy

## Abstract

**Introduction:**

Mobile Schwannoma is a rare soft tissue tumor that commonly involves the elderly population. It has no cellular material and grows as solitary, firm, oval, encapsulated benign tumors from the sensory (dorsal) nerve root. If multiple, they are usually associated with Neurofibromatosis type 2 (NF-2). The initial sign and symptoms include segmental pain and paresthesia. It may lead to myelopathy if the tumor expands.

**Case presentation:**

We present a twenty-year-old female with chronic lower backache radiating to the ipsilateral thigh with no urinary or fecal incontinence. On physical examination, the ankle reflex was hypoactive on the left side, and the straight leg raise test was positive. A large 32 × 15 × 14 mm heterogeneous enhancing focal lesion was found on the posterior side of L5 and S1 vertebrae with severe central canal stenosis. A diagnosis of nerve sheath tumor was made based on contrast MRI pre-operatively. The underlying cause was a nerve sheath tumor. A total bilateral laminectomy at the L1-S5 level and mass excision was performed, preserving nerve roots. The postoperative period was uneventful, and no tumor re-growth was noticed.

**Clinical discussion:**

Schwannoma is a slow-growing tumor; benign; usually, less than 8 cm in diameter tumor, commonly found in the head and neck region. It is the 3rd most common soft tissue tumor and the 2nd most common intradural extramedullary tumor. In our report, a young, non-Caucasian female patient is diagnosed with schwannoma, which is quite rare. In our case, a larger tumor of size 32 × 15 × 14 mm was noted, affecting the posterior body of L5 and S1 vertebrae in the left lateral recess with impingement of the left S1 traversing nerve root. Around 29% of spinal root nerve tumors are schwannomas. As the tumor grows slowly, the diagnosis may be delayed.

**Conclusion:**

Schwannoma is a slow-growing solitary, firm, oval, encapsulated benign tumor arising from the sensory (dorsal) nerve root. Histopathology plays a vital role in diagnosis, and overall, the disease has a favorable prognosis. Therefore, an appropriate approach is necessary to rule out the disease.

## Highlights

• Schwannomas are benign, solitary, firm tumors. Primarily occur in the fourth and sixth decades of life.
• It is most commonly associated with neurofibromatosis type 2 (NF-2) in children.
• WHO defines spinal schwannoma as a benign grade 1 nerve sheath tumor that arises from Schwan cell.
• Histopathology plays a vital role in diagnosis, and overall, the disease has a favorable prognosis.

## 1 Introduction

Schwannomas are also known as neurilemmomas or neurinomas. They contain no cellular material and grow as solitary, firm, oval, encapsulated benign tumors from the sensory (dorsal) nerve root [1]. Schwannomas primarily occur in the fourth and sixth decade of life and are associated with the 8th cranial nerve or the spinal nerves [2]. Multiple schwannomas are associated with Neurofibromatosis type 2 (NF-2) with a higher incidence and recurrence rate [3]. Initial symptoms include segmental pain and paresthesia. If the tumor expands, it can eventually lead to myelopathy [4]. This report presents a 20years old patient with a surgically treated large localized schwannoma of the lumbar spine. This is the first case report of such a large schwannoma of the lumbar spine to the best of our knowledge.

This case report has been reported in line with the SCARE Criteria [5].

## 2 Case presentation

A 20-year-old previously well female presented with progressive, severe lower backache for four years, with recent radiation to the left lower limb. The pain was relieved by rest and analgesics, aggravated on exertion, and standing long. She did not have urinary or fecal incontinence. The patient's family history was unremarkable.

On physical examination, the ankle reflex was hypoactive on the left side, and the straight leg raise test was positive. The patient had hyperalgesia in S1 dermatomal distribution. The anal tone was normal, with no perianal numbness. The rest of her examination was unremarkable. Her routine laboratory investigations were normal, including complete blood count, inflammatory markers, electrolytes, renal function, and liver function tests.

A provisional diagnosis of spinal nerve compression was made. An urgent contrast-enhanced lumbosacral magnetic resonance imaging (MRI) was ordered. The MRI report revealed diffuse disc bulge at the L5-S1 level, causing indentation on the thecal sac. A large 32 × 15 × 14 mm heterogeneous enhancing focal lesion was found on the posterior side of L5 and S1 vertebrae in the left lateral recess with impingement of the left S1 traversing nerve root (Fig. 1a) with severe central canal stenosis. The underlying cause was a nerve sheath tumor (Fig. 1b).

**Fig. 1a:**
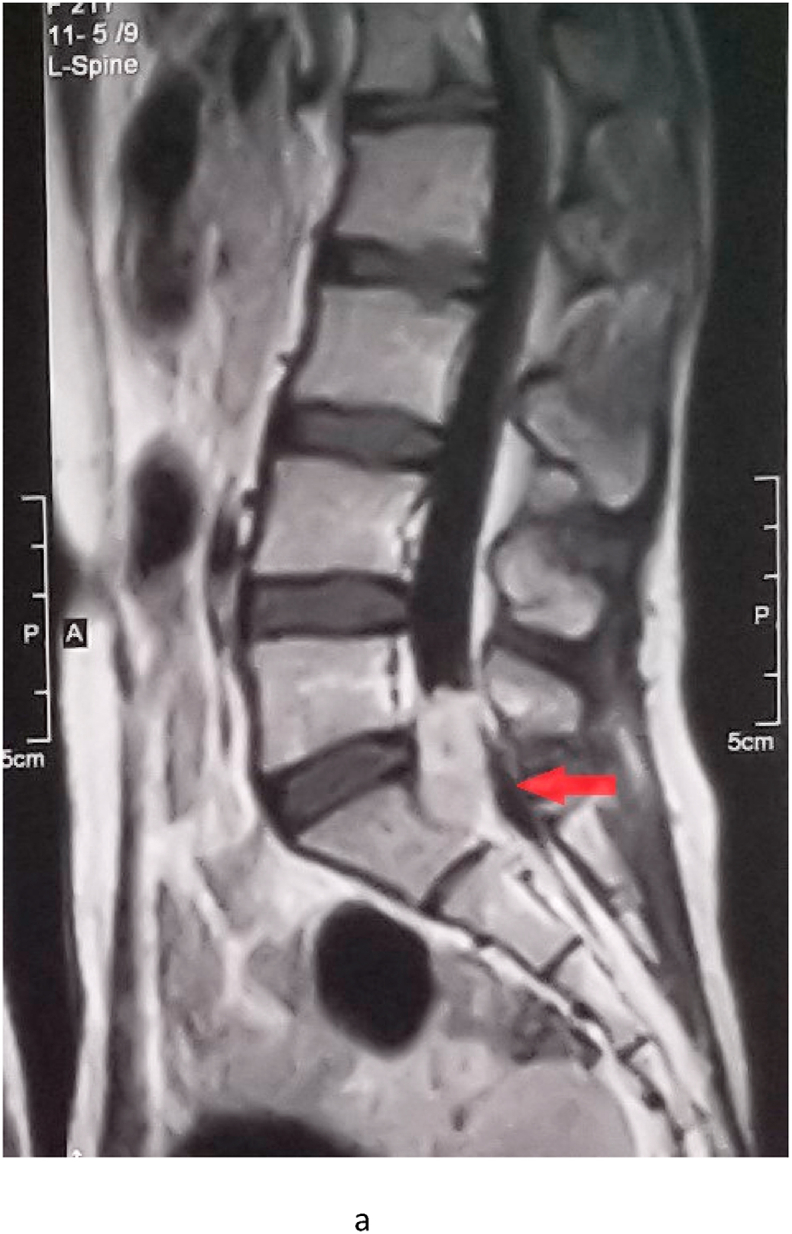
Red arrow shows a lesion on the posterior side of L5 and S1 vertebrae in the left lateral recess with impingement of the left S1 traversing nerve root. (For interpretation of the references to colour in this figure legend, the reader is referred to the Web version of this article.)

**Fig. 1b:**
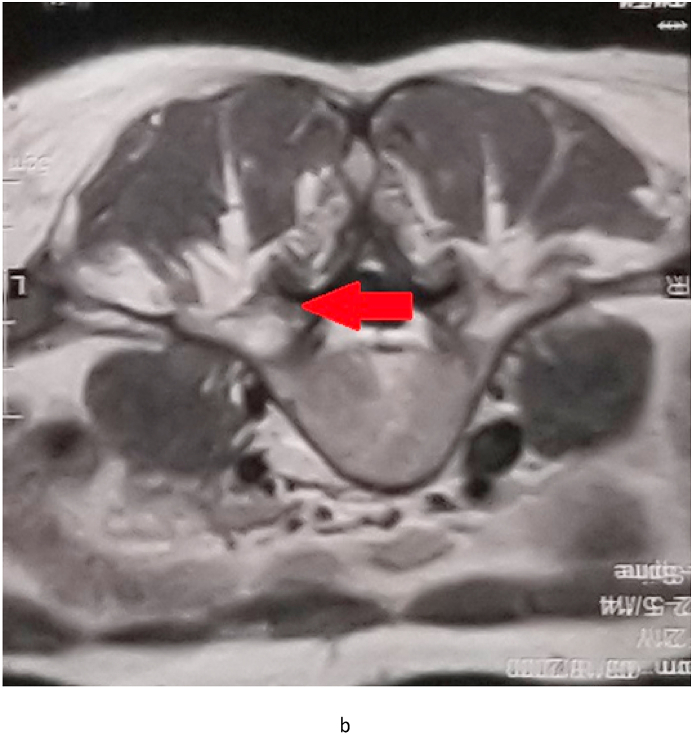
In Axial MRI, the red arrow shows a lesion on the posterior side of the vertebra with severe central canal stenosis. (For interpretation of the references to colour in this figure legend, the reader is referred to the Web version of this article.)

A multi-disciplinary team meeting was done, and surgery was planned to preserve nerve root and sympathectomy as a permanent relief solution. The surgery was done under general anesthesia. The patient was placed in the prone position; after prepping and draping with povidone-iodine, an 8 cm skin incision was made in the Lumbosacral area (Fig. 2a). We reached the L5-S1 level by dissecting subcutaneous tissue and paraspinal muscle, maintaining the hemostasis with bipolar. Bilateral total laminectomy at L5-S1 was performed. A large mass was removed, preserving the nerve roots shown in Fig. 2b. The area was fixed with four titanium screws and titanium rods placed at L5 and S1 vertebrae (Fig. 3). The tissue was repaired plane by plane. After surgery patient was transferred to the neurosurgery intensive care unit for the continuation of care. The recovery was uneventful.

**Fig. 2a:**
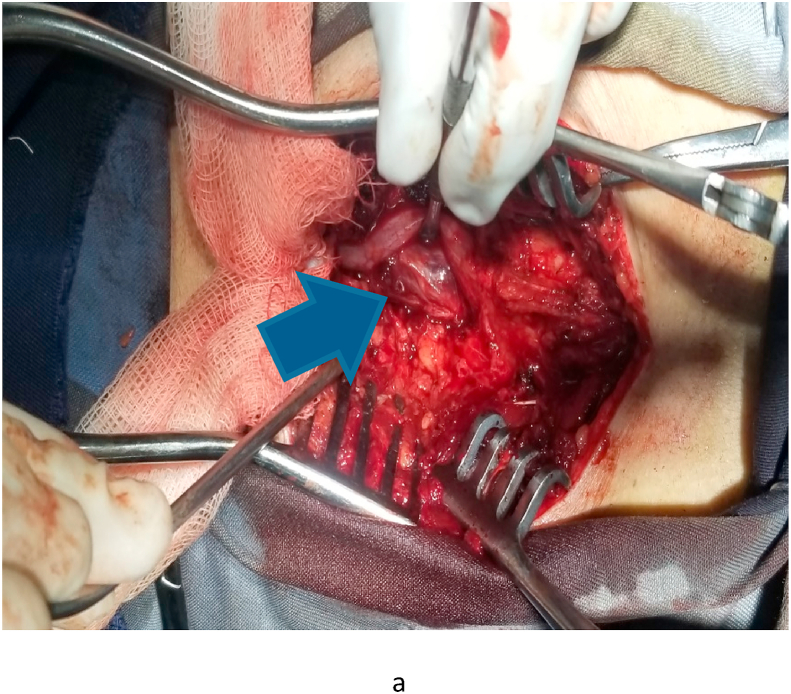
Intraoperative (total bilateral laminectomy of L5-S1 the arrow indicates the lesion.

**Fig. 2b:**
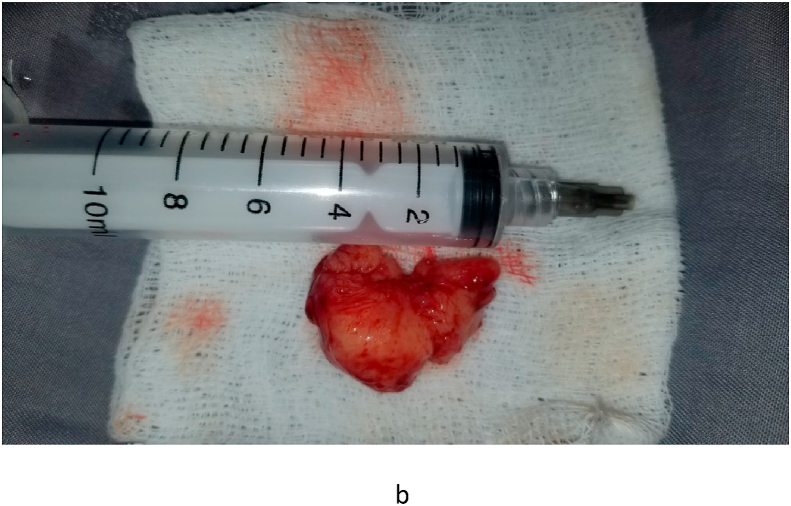
Intraoperative mass.

**Fig. 3:**
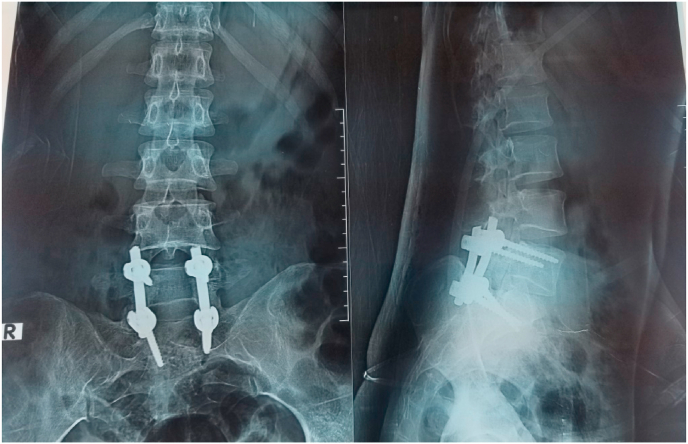
Postoperative X-ray of the patient (fixed with screw and rods).

We achieved total resection of the nerve sheath tumor without any postoperative neurological deficit. After surgery, the patient was hospitalized for a week. The backache resolved completely. The patient is following up in the neurosurgery clinic. Postoperative histopathology of the mass revealed schwannoma. 6-week follow-up showed no radiologic evidence of disease recurrence.

## 3 Discussion

Schwannoma is the 3rd most common soft tissue tumor and the 2nd most common intradural extramedullary tumor, followed by spinal meningioma [6]. It is a slow-growing tumor; usually, less than 8 cm in diameter, most commonly found in the head and neck region [7]. Ahmet Öğrenci et al. reported that schwannoma predominates in Caucasian males, with the peak incidence of 65–74 years. We reported a young, non-Caucasian female patient, which is rare [8].

World Health Organization (WHO) defines spinal schwannoma as a benign grade 1 nerve sheath tumor that arises from Schwan cell [8]; 29% of spinal root nerve tumors are schwannomas. As the tumor grows slowly, the diagnosis may be delayed. Low backache is the initial feature. As the tumor grows, symptoms evolve and can lead to dysesthesia and autonomic dysfunction [9]. Retroperitoneal growth of the tumor can lead to abdominal pain, weight loss, and other atypical symptoms, including headaches, renal colic, and hematuria [6].

Hagiwara et al. reported 20 × 15 × 12 cm tumor in the buttock [9]. In our case, a larger tumor was noted, affecting the posterior body of L5 and S1 vertebrae in the left lateral recess with impingement of the left S1 traversing nerve root.

Local bony changes caused by the schwannoma growth are usually visible in X-ray, CT, and MRI examinations [10,11]. These changes include destruction of the vertebral pedicle, dilation of the vertebral canal, changes in the vertebral body, and an increase in the distance between vertebral pedicles. Schwannomas may result in pressure erosion in nearby bony structures [12]. Our patient did not have any widespread osseous changes, but severe central canal stenosis was seen. 1% of schwannomas can become malignant per year [13]. One per 1000 cases transformed to a malignant tumor, and 76% of tumors are usually 5cm in size [13]. Our patient had no malignant changes or evidence of metastasis.

## 4 Conclusion

Schwannoma is a slow-growing solitary, firm, oval, encapsulated benign tumor arising from the sensory (dorsal) nerve root. It is most commonly associated with neurofibromatosis type 2 (NF-2) in children. Schwannoma of the lumbosacral area is a rare but distinct entity of benign tumor of intra-dural/soft tissue tumors and can lead to chronic back pain. Solitary schwannomas without neurofibromatosis are rare. Histopathology plays a vital role in diagnosis, and overall, the disease has a favorable prognosis.

## Patient perspective

The patient did not present his point of view.

## Source of funding

The authors declare that they have no funding.

## Provenance and peer review

Not commissioned, externally peer-reviewed.

## Ethical approval

This is a case report that does not require formal ethical committee approval.

## Ethical approval

NA.

## Sources of funding

None.

## Conflicts of interest

None.

## Registration of research studies

Name of the registry: NA.

Unique Identifying number or registration ID: NA.

Hyperlink to your specific registration (must be publicly accessible and will be checked): NA.

## Guarantor

Irfan Ullah.

Kabir Medical College, Gandhara University, Peshawar, Pakistan. Irfanullahecp2@gmail.com.

+923340968239.

## Consent

Yes.

## Declaration of competing interest

The authors declare that there is no conflict of interest.

## References

[1] Rodriguez F.J., Folpe A.L., Giannini C., Perry A. (2012). Pathology of peripheral nerve sheath tumors: diagnostic overview and update on selected diagnostic problems. Acta Neuropathol..

[2] Fehlings M.G., Nater A., Zamorano J.J., Tetreault L.A., Varga P.P., Gokaslan Z.L., Chou D. (2016). Risk factors for recurrence of surgically treated conventional spinal schwannomas: analysis of 169 patients from a multicenter international database. Spine.

[3] Alexiev B.A., Chou P.M., Jennings L.J. (2018). Pathology of melanotic schwannoma. Arch. Pathol. Lab Med..

[4] Adogwa O., Fessler R.G. (2017). Brain and Spine Surgery in the Elderly.

[5] Agha R.A., Franchi T., Sohrabi C., Mathew G., for the SCARE Group (2020). The SCARE 2020 guideline: updating consensus surgical CAse REport (SCARE) guidelines. Int. J. Surg..

[6] Jankowski R., Kościński J., Sokół B., Malinger S., Szymaś J. (2015). Presacral schwannoma. Case description. J. Med. Sci..

[7] Tish S., Habboub G., Lang M., Ostrom Q.T., Kruchko C., Barnholtz-Sloan J.S., Kshettry V.R. (2019). The epidemiology of spinal schwannoma in the United States between 2006 and 2014. J. Neurosurg. Spine.

[8] Öğrenci A., Koban O., Şentürk S., Yaman O., Sasani M., Dalbayrak S. (2017). Giant spinal schwannomas. Clin Surg.

[9] International Agency for Research on Cancer (2016).

[10] Nanda A., Kukreja S., Ambekar S., Bollam P., Sin A.H. (2015). Surgical strategies in the management of spinal nerve sheath tumors. World neurosurgery.

[11] Lenzi J., Anichini G., Landi A. (2017). Spinal Nerves schwannomas: experience on 367 cases-historic overview on how clinical, radiological, and surgical practices have changed over a course of 60 years. Neurol. Res. Int..

[12] Chen H., Xu Q., Zhan P., Liu Y., Dai M., Zhang B. (2019). Giant paravertebral schwannoma near the lumbar nerve roots with bone destruction: a case report. Medicine.

[13] Ando K., Imagama S., Ito Z., Kobayashi K., Yagi H., Hida T. (2016). How do spinal schwannomas progress? The natural progression of spinal schwannomas on MRI. J. Neurosurg. Spine.

